# Studying the Contribution of Serotonin to Neurodevelopmental Disorders. Can This Fly?

**DOI:** 10.3389/fnbeh.2020.601449

**Published:** 2021-01-12

**Authors:** Angel Carvajal-Oliveros, Jorge M. Campusano

**Affiliations:** ^1^Departamento de Genética del Desarrollo y Fisiología Molecular, Instituto de Biotecnología, Universidad Nacional Autónoma de México, Cuernavaca, Mexico; ^2^Laboratorio Neurogenética de la Conducta, Departamento de Biología Celular y Molecular, Facultad de Ciencias Biológicas, Pontificia Universidad Católica de Chile, Santiago, Chile; ^3^Centro Interdisciplinario de Neurociencia UC, Pontificia Universidad Católica de Chile, Santiago, Chile

**Keywords:** neurodevelopmental disorders, serotonin, SERT, *Drosophila*, ASD

## Abstract

Serotonin is a biogenic amine that acts as neurotransmitter in different brain regions and is involved in complex behaviors, such as aggression or mood regulation. Thus, this amine is found in defined circuits and activates specific receptors in different target regions. Serotonin actions depend on extracellular levels of this amine, which are regulated by its synthetic enzymes and the plasma membrane transporter, SERT. Serotonin acts also as a neurotrophic signal in ontogeny and in the mature brain, controlling cell proliferation, differentiation, neurogenesis, and neural plasticity. Interestingly, early alterations in serotonergic signaling have been linked to a diversity of neurodevelopmental disorders, including autism spectrum disorder (ASD), attention deficit/hyperactivity disorder (ADHD), or mental illnesses like schizophrenia or depression. It has been proposed that given the complex and numerous actions of serotonin, animal models could better serve to study the complexity of serotonin actions, while providing insights on how hindering serotonergic signaling could contribute to brain disorders. In this mini-review, it will be examined what the general properties of serotonin acting as a neurotransmitter in animals are, and furthermore, whether it is possible that *Drosophila* could be used to study the contribution of this amine to neurodevelopmental and mental disorders.

## Serotonin is a Biogenic Amine Acting Not Only as a Classical Neurotransmitter, But also as a Neurotrophic Factor

Biogenic amines (BAs) are a group of neuroactive molecules that contain one or more amino group, are synthesized from amino acids, and act as classical neurotransmitters, neuromodulators or neurohormones. Among them, serotonin is a BA associated with a number of physiological processes and the control of several behaviors, including sleep regulation, social rank, mood and learning. The alteration of serotonergic neural systems is associated with some neurodevelopmental and mental disorders, including anxiety, depression and ASD. However, we are far from fully understanding the complex cellular and molecular actions of amines and how the alteration of neural systems that store and release them contributes to these disorders.

**FIGURE 1:**
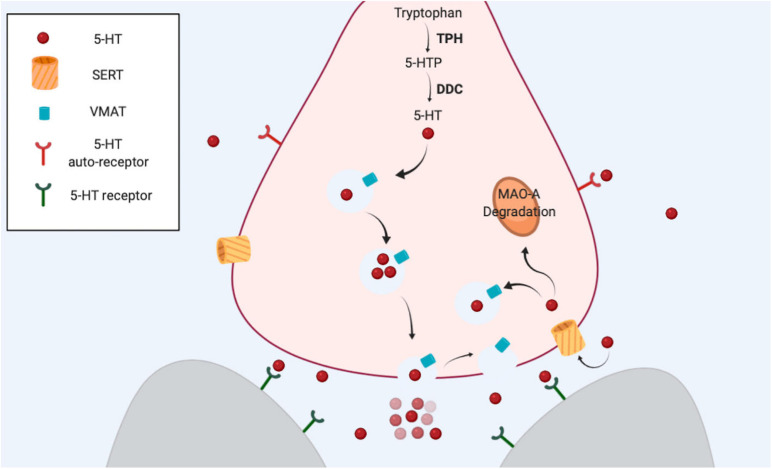
The serotonergic synapse. Serotonin is synthetized from amino acid tryptophan. The first step is the hydroxylation of tryptophan carried out by TPH, the limiting enzyme in this process. DDC catalyzes the final step. VMAT transport serotonin from the cytosol into the vesicles. After an action potential reaches the terminal, serotonin is released from vesicles, and the neurotransmitter is able to bind and activate specific receptors in the same neuron (presynaptic) or in other neurons (postsynaptic). SERT reuptakes serotonin back to the cytosol in the presynaptic neuron. The amine can be used again as a neurotransmitter or alternatively can be degraded by MAO-A.

As with other classical neurotransmitters, serotonin is synthetized in the cytosol in a two-step biosynthetic pathway (Figure 1). The enzyme tryptophan hydroxylase (TPH, aka TRH or TrpH) catalyzes the conversion of Tryptophan to 5-hydroxytryptophan. Then, the Dopa decarboxylase enzyme (DDC, aka AADC) catalyzes the final conversion to serotonin (5-hydroxytryptamine). Serotonin is stored in vesicle compartments located mainly in axon terminals (the presynaptic neuron). Upon the arrival of an action potential, these vesicles fuse with the plasma membrane to release their content in the extracellular space (the synaptic cleft), so that the neurotransmitters can reach specific receptors in the postsynaptic neuron to induce cellular responses. These receptors mostly belong to the superfamily of G protein-coupled metabotropic receptors. Finally, the end of the action of the neurotransmitter depends on the reuptake of the chemical from the synapse back into the presynaptic neuron, via SERT. Once inside the cytosol, the neurotransmitter can be reutilized as a neurotransmitter (is transported back into a synaptic vesicle by the vesicle monoamine transporter, VMAT), or it can be metabolized by the MAO enzyme (Daubert and Condron, 2010). The two biosynthetic enzymes and SERT help define the neurochemical identity of a neuron as serotonergic (Figure 1).

**FIGURE 2:**
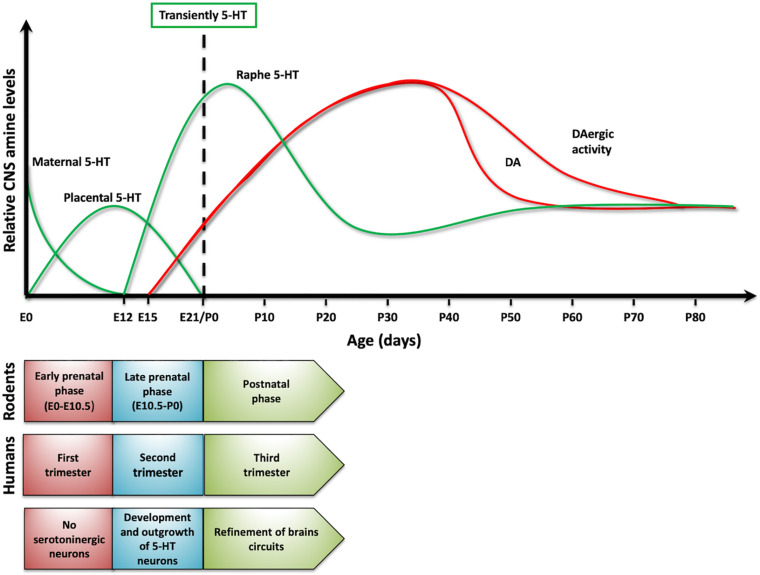
Serotonin dynamics on development. The **upper panel** presents changes in serotonin levels (green traces) in rodents and its sources. As a comparison, it presented dopamine levels (red traces). In **lower panel**, it compared developmental stages in rodents and humans, as serotonin levels change over development.

Interestingly, serotonin also plays a critical role as a signaling/trophic molecule early in development (Gaspar et al., 2003; Daubert and Condron, 2010; Bonnin and Levitt, 2011). Actually, when no cells from the developing organism are able to synthetize or release serotonin, some placental cells transiently acquire the ability to synthetize and release serotonin, which can then reach the embryo (Bonnin and Levitt, 2011). Earlier, the first source for serotonergic information for the rodent embryo is the maternal blood (Koren et al., 1966). In addition, neurons that in the rodent mature brain are not serotonergic (e.g., thalamic neurons), transiently express VMAT and SERT early in development (by E13) in order to accumulate and release serotonin at later times (Lebrand et al., 1996; Cases et al., 1998). All these findings support the idea that this amine is required early in development and that non-embryonic sources of this BA are crucial to fulfill this need. Later in development, the embryonic neurons of the Raphe Nucleus, the main serotonergic nucleus in vertebrates, acquire the ability to synthetize and release serotonin, and the fetus becomes independent of the exogenous supply of the amine. It is possible to find the first serotonergic neurons in 5-week old human embryos, earlier than other aminergic populations (Sundstrom et al., 1993). A similar situation—serotonin neurons established a little earlier than dopaminergic cells—is observed in rodents. Thus, it is possible to propose a dynamic change in serotonin levels in the central nervous system (CNS) over development (Figure 2; Suri et al., 2015). Importantly, it has been postulated that interruption or alteration of the serotonergic information reaching the embryo or fetus, is implicated in a higher incidence for several brain disorders including ASD, major depressive disorder (MDD), ADHD, anxiety, and schizophrenia, among others (Schain and Freedman, 1961; Bleich et al., 1988; Caspi et al., 2003; Hranilovic et al., 2007; Olivier et al., 2011). This mini review intends to discuss the contribution of serotonin to the onset and progression of behavioral traits common to several neuropsychiatric diseases. However, particular attention is placed on ASD, given that several evidences support that this amine plays a role in this particular disorder.

## Alteration in Serotonin Levels Associated With ASD and Other Brain Disorders

It has been reported that about 30% of children diagnosed with ASD show higher levels of serotonin in the blood as compared to control children (Gabriele et al., 2014). The increased serotonemia seems to be unrelated to any specific genetic alteration linked to this disorder. Actually, only about 15–20% of ASD cases are genetic; among the alterations associated with ASD, it has been reported mutations in specific genes (e.g., Shank or Neuroliguin 3), gene copy number variation, and chromosomal disorders (Miles, 2011). Increase in blood serotonin concentration is also detected in schizophrenic patients, while hyposerotonemia has been described in people diagnosed with depression (Muck-Seler et al., 2004). Most of these disorders are classified as neurodevelopmental disorders, in that genetic or genomic alterations underlying these conditions are present as early as the embryo develops and/or over critical time windows in the development of brain neural circuits (Suri et al., 2015).

Similarly, it has been proposed that environmental factors affecting serotonergic signaling (e.g., exposure to drugs affecting the serotonergic system) could play a role in neurodevelopmental disorders. It has been shown in rodent models that these chemicals could be particularly effective at hindering serotonergic signaling early over development, since the blood–brain barrier (BBB) becomes fully functional only by E15 (Ben-Zvi et al., 2014). Therefore, at earlier time points there is no obstacle that stops these chemicals from acting at targets such as serotonin receptors or SERT, affecting serotonergic signaling both in the periphery and centrally. SERT plays a crucial role as the major regulator of serotonin homeostasis and over recent years this transporter has been the focus of several studies (reviewed in Muller et al., 2016). Importantly, several environmental stimuli modifying SERT operation and some mutations affecting the SERT gene have been associated to brain disorders, including ASD.

## Gene and Environmental Alterations Affecting SERT, Linked to Brain Disorders

### Mutations in SERT Linked to Neurodevelopmental and Mental Disorders

Several modifications in the gene coding for SERT have been associated to different brain disorders, including obsessive-compulsive disorder and Asperger syndrome/ASD (Kilic et al., 2003; Prasad et al., 2009). Some are located in the untranslated regions (UTR) of SERT gene, particularly the promoter, resulting in decreased transported expression. Reduced SERT expression could underlie the increased extracellular serotonin levels found in ASD and other disorders. Interestingly, some of the SERT mutations described (e.g., Ile425Leu, Phe465Leu, and Leu550Val) are gain-of-function mutations that increase the activity of the transporter, but hinder SERT insertion in the plasma membrane. Thus, reduced localization of SERT in the membrane would result in reduced serotonin uptake and consequently hyperserotonemia (Prasad et al., 2009), as it is reported in ASD (Gabriele et al., 2014).

### Environmental Manipulations Affecting SERT and Linked to Brain Disorders: Clinical Studies

It is estimated that about 10% of pregnant women are prescribed antidepressant drugs for treatment of clinical depression and anxiety (Cooper et al., 2007; Huybrechts et al., 2013). A big proportion of antidepressant drugs are Serotonin Selective Reuptake Inhibitors (SSRIs), whose molecular target is SERT, and include fluoxetine and citalopram (Patil et al., 2011). These chemicals are able to cross cell membranes and tissues like the placenta and the BBB (Heikkinen et al., 2002), which also explains why that they can be found in the breast milk (Hendrick et al., 2001). The focus of this mini-review is on discussing how manipulations affecting SERT result in brain-associated deficits, but several reports have described that prenatal exposure to SSRIs is associated to increased risk for preterm delivery (Davis et al., 2007; Maschi et al., 2008; Reis and Kallen, 2010; Huang et al., 2014), low birth weight (Oberlander et al., 2006; Reis and Kallen, 2010; Huang et al., 2014), and cardiac defects (Kallen, 2007; Pedersen et al., 2009; Kornum et al., 2010). This is consistent with the idea that serotonin plays key roles in the development of the entire organism.

A number of reports have argued that prenatal exposure of SSRIs is associated to higher incidence of ASD (e.g., Boukhris et al., 2016). Similar studies have shown a positive correlation between prenatal SSRI exposure and ADHD (Figueroa, 2010; Clements et al., 2015). Only one study (Malm et al., 2016) has assessed depression and prenatal exposure to SSRIs, and reported a positive correlation, as well. On the other hand, a different set of studies has argued that no association between prenatal exposure to SSRIs and ASD or ADHD exists (e.g., Castro et al., 2016). Interestingly, this and other studies (Mezzacappa et al., 2017) proposed that a relevant factor explaining the higher incidence for ASD is the medical condition of the mother—i.e., maternal depression. However, Croen et al. (2011) determined no increase in the risk of ASD in the offspring of mothers with a history of depression, an effect that did not depend on prenatal consumption of SSRI. All these (and other) studies (Kaplan et al., 2017; Maloney et al., 2018; Halvorsen et al., 2019) show that it is not clear whether the use of SSRIs results in increased incidence for ASD, ADHD or any other disorder. One additional problem with these studies is the difficulty to access and study larger populations (as discussed in Millard et al., 2017). In spite of these considerations, it is possible to argue that SSRIs could reach the embryo and neonates and possibly affect the developing central nervous system in a way not fully understood but that could lead to brain disorders. In reviewing the available information on this issue, authors have come to the conclusion that relevant information to further support or discard the contribution of SSRIs to these disorders could be obtained from animal models (Pedersen, 2015).

### Environmental Manipulations Affecting SERT and Linked to Brain Disorders: Animal Models

When modeling human disorders in animals, researchers have focused on replicating one or few specific behavioral features, although it has to be considered that some features are difficult to recreate in animal models (e.g., hallucinations or psychosis) (Anderson and Adolphs, 2014). Thus, for instance, ASD animal models usually recreate repetitive behaviors or the impaired social interaction observed in this disorder (Yenkoyan et al., 2017). An additional issue to be considered when modeling these disorders is that it is estimated that postnatal day 7 in rodents is equivalent to time of birth in humans (Figure 2). This means that a postnatal manipulation in rodents could be equivalent to a prenatal one in humans (Clancy et al., 2007). In spite of these caveats, the key impact of animal models is on advancing our understanding of these disorders at the cellular, molecular, neurochemical and/or circuit levels.

In particular, very few studies have directly assessed the possibility that antenatal or perinatal exposure to SSRIs affects incidence of ASD-like features in animals. For instance, Sprowles et al. (2016) studied the effects of perinatal exposure to citalopram in rats. Results obtained demonstrate several autistic-like behavioral traits in the offspring (repetitive behaviors, impaired social behavior), which are consistent with the concurrent description of anatomical alterations in Raphe and in cortical structure and physiology (Darling et al., 2011; Simpson et al., 2011). These findings argue in favor of the idea that prenatal SSRI exposure increases the incidence of ASD. A different study reported that perinatal inhibition of MAO, which could be considered a manipulation that like SSRIs increases the availability of serotonin, results in alteration of serotonin metabolism and hyperserotonemia (Hranilovic et al., 2011), and an increased incidence of ASD features (Davis et al., 2008). There is not much information on how perinatal exposure to SSRIs affects incidence for other disorders, and the little data available is contradictory. For instance, while one report supports the idea that perinatal exposure to SSRIs exacerbates depressive-like behaviors in a strain of rats prone to anxiety and depression (Glover et al., 2015), other works propose that perinatal SSRIs partially reverses some of these behavioral traits (Rayen et al., 2011; Boulle et al., 2016), while another study shows no effect of the treatment (Zohar et al., 2016). Unfortunately, these studies have not been consistent with the SSRI used, doses of these chemicals, the developmental age at which interventions begin, or the gender of the animals studied, among other factors. Thus, it remains an open question whether exposure to SSRIs over ontogeny contributes to brain disorders.

On the other hand, increased serotonin levels observed in SERT knockout mice have been associated with abnormal development of thalamocortical axons and somatosensory cortical barrels (Persico et al., 2001; Gaspar et al., 2003), and also anxiety and depressive-like behaviors (Ansorge et al., 2004).

## *Drosophila* as Animal Model for the Study of Neurodevelopmental and Mental Disorders

The vinegar fly *Drosophila melanogaster* has served as a workhorse in various fields in biology, in part based on the diverse genetic toolbox available, as discussed elsewhere in this special issue. Remarkably, *Drosophila* exhibits a complex behavioral repertoire. For instance, one of the best-studied social behaviors in flies is courtship behavior: male flies court a female animal in order to mate, a behavior that is decreased after males have experienced rejection by a fertilized female (Siegel and Hall, 1979; Kamyshev et al., 1999). New social paradigms have been described including the study of clustering behavior observed in groups of flies (Simon et al., 2012). Recent studies have also shown that flies may exhibit attention-like processes (van Swinderen and Flores, 2007), goal-driven behavioral adaptations (Pick and Strauss, 2005) and decision making (Zhang et al., 2007). *Drosophila* has been also been used as a model organism to study aggression (Baier et al., 2002) and addiction (Wolf, 1999).

In recent years, it has become evident that it is possible to model neurodevelopmental disorders in *Drosophila*, aiming at reproducing some of the key behavioral traits associated with these illnesses. One of the best-studied models for neurodevelopmental disorders in *Drosophila* is the ASD model based on mutations in the FMR1 gene (van Alphen and van Swinderen, 2013). This is a gene associated with fragile X syndrome, a disorder linked to intellectual disability and where a high incidence for ASD is reported. *Drosophila FMR1* mutants exhibit reduced memory in a courtship social paradigm (McBride et al., 2005) and repetitive grooming, which is reminiscent of recurring behaviors observed in ASD (Tauber et al., 2011). In addition, brain and circuit organization is affected in fly *FMR1* mutants (Siller and Broadie, 2011), consistent with structural changes in axonal and dendritic branches, a feature shared by mice FMR1 mutants (Zhang et al., 2001; Zhang and Broadie, 2005; Callan and Zarnescu, 2011; van Alphen and van Swinderen, 2013).

### *Drosophila* as an Animal Model for Studying the Contribution of Serotonin to Neurodevelopmental and Mental Disorders?

The molecular mechanisms involved in serotonin biosynthesis are evolutionary conserved, and in *Drosophila*, they begin with the hydroxylation of the tryptophan amino acid by TPH (Coleman and Neckameyer, 2005). Likewise, it has been described a *Drosophila* SERT (Giang et al., 2011; Hidalgo et al., 2017) and one VMAT (Greer et al., 2005) that share structural and functional similarities to that of vertebrates. Five serotonin receptors have been described in the *Drosophila* genome, all of them classified as metabotropic. Thus, the *Drosophila* serotonergic system is highly conserved as compared to its mammalian counterpart (Kasture et al., 2018). Importantly, it is already known that serotonin contributes to several behaviors in *Drosophila* including locomotion, feeding behavior, circadian activity, sleep regulation, and aggression (Silva et al., 2014; Kasture et al., 2018; Bacque-Cazenave et al., 2020).

Out of the approximately 100,000 neurons in the fly brain, about 80 cells are identified as serotonergic neurons, organized in 11 clusters (reviewed in Kasture et al., 2018)). Valles and White (1988), by using immunochemistry, described the serotonergic neural system in the larval and adult CNS and also described how serotonin levels change over development. The first detection of serotonin-positive cells is in 16–20 h *Drosophila* embryos. The detection of immunopositive serotonin cells before fly CNS is fully developed and supports the idea that serotonin could play a role as developmental signaling molecule, as in vertebrates (Lundell and Hirsh, 1994). In this regard, it has been suggested that serotonin modulates the development of serotonergic varicosities in the fly CNS (Sykes and Condron, 2005). Consistent with this, mutants in DOPA decarboxylase which are associated with reduced amine levels exhibit alterations in branch spacing (Budnik et al., 1989). Conversely, overexpression of TPH in *Drosophila* promotes higher levels of cytoplasmic serotonin, which is related with abnormalities in neurite morphology in larval and adult fly neuropils (Daubert et al., 2010). Moreover, altering serotonin synthesis in early embryos results in impaired anatomy and functioning of the feeding circuit in larvae, a phenotype that can be reversed as serotonin levels are rescued (Neckameyer, 2010). All these findings suggest that, as in mammals, hindering serotonergic signaling at early developmental stages does have implications for the establishment of mature circuits that underlie behaviors. However, the information on this issue is limited. New research should ask whether pharmacological or genetic manipulations tampering with serotonergic components (SERT, biosynthetic enzymes or any of the receptors), affect the organization of brain circuits and consequently, result in behavioral features associated with neurodevelopmental or mental disorders.

Although the literature has not thoroughly explored this, there are some reports supporting this idea. For instance, centrophobism, a behavior in which flies avoid the center of an arena, is considered to be an anxiety-like behavior in flies. Different genetic or pharmacological manipulations that affect SERT functioning affect centrophobism in *Drosophila* (Mohammad et al., 2016; Hidalgo et al., 2017). In particular, we showed that feeding flies an amphetamine derivative that stimulates serotonin release decreases centrophobism, a similar result observed in animals mutant for SERT (Hidalgo et al., 2017). In addition, a recent work from our group showed deficits in social behavior and locomotion in a *Drosophila* mutant for the dysbindin gene, an animal model for schizophrenia. Interestingly, the phenotypes observed in the dysbindin mutants seem to depend at least in part on altered serotonergic signaling (Hidalgo et al., 2020).

Demonstrating that serotonin signaling is affected in fly models for brain disorders, is not only relevant for advancing our understanding of the underpinnings of these illnesses, it also opens up the possibility to carry out a high-throughput search for new chemicals that affect specific phenotypes in flies, which could eventually lead to new therapeutic tools for these disorders (Nichols, 2006; Roy et al., 2020).

A better understanding of serotonin dynamics over development and how serotonergic deficiency could be involved in mental disorders could provide insights in the search for new treatments for these disorders, a path in which *Drosophila* could play an important role.

## Author Contributions

AC-O and JC wrote this manuscript. Both authors contributed to the article and approved the submitted version.

## Conflict of Interest

The authors declare that the research was conducted in the absence of any commercial or financial relationships that could be construed as a potential conflict of interest.
